# Facing the Spectator

**DOI:** 10.1177/2041669516675181

**Published:** 2016-11-16

**Authors:** Jan Koenderink, Andrea van Doorn, Baingio Pinna, Robert Pepperell

**Affiliations:** University of Leuven (KU Leuven), Belgium; Utrecht University, Netherlands; University of Sassari, Italy; Cardiff Metropolitan University, Cardiff, UK

**Keywords:** pictorial space, picture perception, cue scission, uncanny valley

## Abstract

We investigated the familiar phenomenon of the uncanny feeling that represented people in frontal pose invariably appear to “face you” from wherever you stand. We deploy two different methods. The stimuli include the conventional one—a flat portrait rocking back and forth about a vertical axis—augmented with two novel variations. In one alternative, the portrait frame rotates whereas the actual portrait stays motionless and fronto-parallel; in the other, we replace the (flat!) portrait with a volumetric object. These variations yield exactly the same optical stimulation in frontal view, but become grossly different in very oblique views. We also let participants sample their momentary awareness through “gauge object” settings in static displays. From our results, we conclude that the psychogenesis of visual awareness maintains a number—at least two, but most likely more—of distinct spatial frameworks simultaneously involving “cue–scission.” Cues may be effective in one of these spatial frameworks but ineffective or functionally different in other ones.

## Introduction

**Figure 1.**
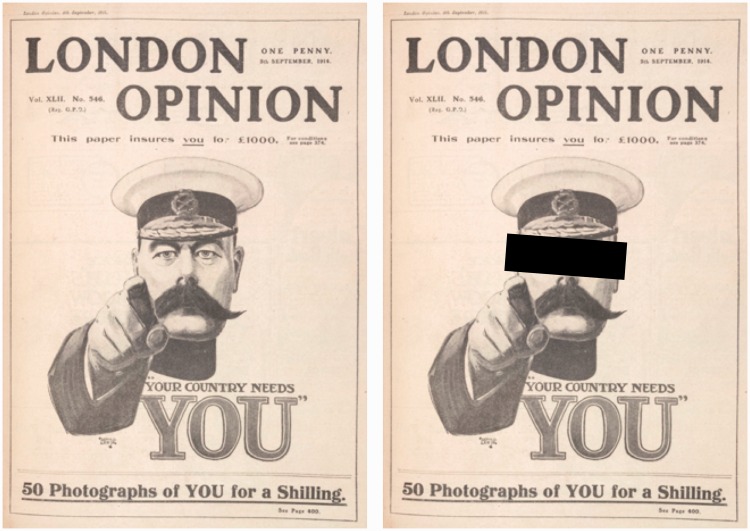
At left, Alfred Leete's (1882–1922) war propaganda poster featuring Lord Kitchener. The impression that the spectator is “directly addressed” from any view point is considered “spooky” by many observers. At right, Lord Kitchener is “facing” the spectator, being deprived of his gaze. Gazing and facing are distinct things.

In a previous, rather extensive, study (*Pointing out of the picture*; Koenderink, van Doorn, Kappers, & Todd, 2004), we have quantified various perceptual aspects of the type that strikes one in Figure 1 (Hecht, Boyarskaya, & Kitaoka, 2014; Maruyama, Endo, & Sakurai, 1985; Rogers, Lunsford, Strother, & Kubovy, 2003).

What is remarkable from a phenomenological perspective, sometimes even referred to as “spooky,” is that the portrayed persons “follow you” as you change your vantage point with respect to the object (poster, painting, … ). Of course, they do nothing of the sort, not being real persons at all, but eidola at best. The experience of “following” must be due to your own psychogenesis. Notice that there are two distinct aspects to “following,” namely that the depicted person “faces you” and that the gaze of the depicted person is such that it appears to fixate you. A famous example is the wartime propaganda poster depicting Lord Kitchener (Figure 1).

There is some literature on the spooky effect (Boyarskaya & Hecht, 2012; Boyarskaya, Sebastian, Bauermann, Hecht, & Tuscher, 2015; Busey, Brady, & Cutting, 1990; Todorovic, 2006, 2009), but most of the marginally relevant literature has to do with the deformations experienced when viewing a perspective rendering from the “wrong” vantage point (Cutting, 1986, 1987a, 1987b; Goldstein, 1979, 1987, 1988; Kerzel & Hecht, 1997; Kubovy, 1986; Pirenne, 1970; Rosinski, Mulholland, Degelman, & Farber, 1980; Vishwanath, Girshick, & Banks, 2005; Yang & Kubovy, 1999). Some of the literature on the perception of gaze direction is also relevant (Anstis, Mayhew, & Morley, 1969; Gale & Monk, 2000; Gamer & Hecht, 2007; Todorovic, 2006, 2009; West & Van Veen, 2007; West, 2015; Wollaston, 1824). The “spookiness” is a purely subjective feeling (Cline, 1967; Freud, 1919; Jentsch, 1995; Misselhorn, 2009; Moore, 2012; Rahimi, 2013; Saygin, Chaminade, Ishiguro, Driver, & Frith, 2011; Seyama, 2007). The portrayed person is certainly seen to be a mere object, perhaps a realistic “portrait,” but certainly not “flesh and blood,” yet it appears miraculously animated. This puts it squarely at yonder side of the “uncanny valley” (Borody, 2013; Mori, 1970).

In two earlier studies (Koenderink, van Doorn, de Ridder, & Oomes, 2010; Koenderink et al., 2004), we touched on the phenomenology. In the present study, we attempt to address the issue in more detail, staying as close to the actual facts—which necessarily have to be “first person reports”—as possible. The results from the previous work are highly relevant to the present work. Although we avoided phenomenological accounts, sticking to objective methods, various apparent “open ends” in the present study were actually addressed in the previous one. This includes variations of real (e.g., rotating the monitor) versus pictorially simulated slant, and so forth. We can hardly summarize that here; the interested reader is referred to the previous, very extensive work.

All of the authors are experienced visual observers. We agree largely on our visual awareness of the uncanny effect. Viewing a panel depicting a face staring at you directly, the depicted person apparently keeps facing you no matter where you are with respect to that panel. This is the familiar Lord Kitchener, or often called (right or wrongly) the Mona Lisa effect. We also notice other visual facts that are less often mentioned. These were already noted in our earlier work (Koenderink et al., 2004), but could not be addressed in detail there. These facts appear to us important and deserving of empirical investigation:

*First*, as the obliqueness of the view changes, the portrait does not seem to be rigidly connected to the frame, as it physically is. That is to say, the pigments are rigidly connected to the panel and thus to the frame, but the portrayed face *appears to change shape* whereas the frame does not. The frame is seen to change its spatial attitude, but it is also seen as rigidly moving, whereas the face suffers a *non-rigid deformation*;
*Second*, the Euclidean normal to the panel and the facing direction apparently coincide when viewing the panel from the front, but these directions diverge from each other when the panel is viewed obliquely. This suggests that the panel and the face are experienced as existing in mutually distinct mental spatial frameworks. In the space of the panel, psychogenesis “corrects” for the foreshortening, whereas in the space of the face, the foreshortening is somehow experienced as a deformation. In oblique view, the face appears to grow “leaner.” Thus, we experience a “scission of cues.”

These effects can easily by verified by the reader by watching our video clips put on the publisher's site. Such observations are conceptually most intriguing. Does stereopsis from monocular cues indeed simultaneously maintain distinct but intermeshed spatial frameworks? This is the topic of the present investigation. We pursue it in a study including a fairly large number of naïve observers and smaller groups of experienced observers from the arts and the sciences.

## Methods

We performed two experiments (henceforth denoted “Experiment I” and “Experiment II”). The presentations were rather different in each case: Experiment I involved several short video clip presentations, each followed by a formal questionnaire, whereas Experiment II involved static presentations in which participants interacted with the stimulus through the adjustments of certain “gauge objects” (Koenderink, van Doorn, & Kappers, 1992).

In all cases, the screen subtends 32.7 by 20.1 of visual angle and is viewed from a distance of 57 cm. Observers view the screen binocularly from a fixed head position. They wear their usual optical correction when necessary. These are almost a requirement in the case of naïve observers.

The room is darkened, so the main impression is that of the luminous display, although there is sufficient scattered light in the room that the physical display monitor remains noticeable, but only of subsidiary visual importance. The participants are familiar with the room, have a haptic relation to their chair, and so forth.

In Experiment I, the screen was dark and video clips were presented at its center. The clips were 512 × 512 pixels (11.4∘×11.4∘), presented at 30 fps. There was a dark period before and after the presentation. The participant could proceed to a next phase of the experiment by hitting the space bar after reading a written cue.

The video clips show a frame with certain content, rotating back and forth about a vertical axis. The slant varies linearly back and forth between ±40∘ extremes with a period of about 1.5 s. Observers are invited to watch the display and answer five simple questions immediately afterwards.

The decision to limit the range to ±40∘ was a difficult one. The major effects that we intend to address are much clearer for narrower ranges. In the generic Leete poster display, we see the frame rigidly rotate but the face deform in a fronto-parallel attitude if the obliqueness is less than about 20∘, whereas for larger angles, the face both rotates and deforms. We only selected the higher value because we intended to involve a large number of naïve observers. The angle was limited to 40∘ because the effect disappears for very large angles (Hecht et al., 2014).

Since five questions severely tax the mental abilities of most observers, we familiarize the observers both with the stimuli and with the list of questions before the actual trial. In a briefing section—implemented as a short video clip—the observers read the list of questions followed by the sequence of video clips. Since the questions involve geometrical concepts, these are illustrated after this first briefing run, after which the list of questions and the clips are identically repeated. The briefings can be watched on the publisher's site. They are designed so as to avoid asking “leading questions” or even suggesting answers. As mentioned earlier, the task is considered a hard one by generic participants as evident from freely offered remarks.

It would have been preferable to rerun the video clip for each question, but this was not a possibility given our resources.

In Experiment II, we use pictorial presentations on the flat display, the observer being at a fixed position with respect to the screen. The pictorial content has a flat, brick wall in either frontal or oblique attitude with a panel consisting of a framed picture attached to the wall at the center. The oblique attitudes of both wall and panel are thus pictorially rather than physically given. This is different from various paradigms presented in the literature, but it allows us to implement convenient user interaction. The visual impression is that of a scene viewed from a certain angle. The frame has a width of 3.0∘. The slant of the wall and panel is 40∘ of simulated Euclidean angle (in 3D) either facing the left or the right.

We present gauge objects either to the left or the right of the panel. One type of gauge object is used to quantify the apparent slant of the panel and another one to indicate the apparent direction of gaze and the apparent aspect ratio (“shape”) of the face. The observers control the gauge objects using the arrow keys of a keyboard. They can take their time for their setting, this typically involves a few seconds per setting. The interface was summarily explained in a short instruction video clip at the briefing.

All experiments were programmed in Processing 2+, a variety of java developed at M.I.T. and aimed at visual artists and designers (Reas & Fry, 2010), thus the ideal tool for explorative experimental phenomenology.

Observers were solicited from the University of Giessen (Germany), Cardiff School of Art (UK), and University of Sassari (Italy). The Giessen observers were professional vision researchers in various stages of their career, the Cardiff observers were art students, whereas the Sassari participants might be regarded to be “naïve observers.” In the remainder of the article, we refer to the Sassari group as either “Group A” (short) or “the group of naïve observers,” to the Cardiff group as either “Group B” (short) or “the group of artistically non-naïve observers,” and to the Giessen group as either “Group C” (short) or “the group of scientifically non-naïve observers” (where we mean “vision science” in particular).

There were 166 participants in Group A, 13 participants in Group B, and 25 participants in Group C. Group A was the least controlled, as it took a heroic effort to collect the responses. As expected, we needed to clean the data, which was done fully algorithmically (see later). Participants were selected as involved in the project if they had indeed answered all questions, were likely to have understood the interface, and so forth.

Gender ratios are very unbalanced, Group A is 83% female, Group B is 92% male, and Group C is 58% male. The majority of participants is young. The median age of Group A participants is 21 (interquartile range 20–24, range 18–54), the median age of Group B is 24 (interquartile range 21–24, range 20–48), and the median age of Group C is 30 (interquartile range 27–43, range 24–72). Although group structure is reported here, we detected no influence of age or gender on our results and will not discuss these issues in the article.

We have no data regarding the participants' stereopsis. We think it likely—on the basis of two decennia of experience (Koenderink & van Doorn, 1995, 2012; Koenderink et al., 1992; Koenderink, van Doorn, & Kappers, 1994; Koenderink, van Doorn, Kappers, & Todd, 2001; Koenderink, van Doorn, & Todd, 2009; Koenderink, van Doorn, & Wagemans, 2011)—that the generic population has very significant fractions of members with lacking or anomalous binocular or monocular stereopsis. Unfortunately, this topic has been seriously underresearched and virtually nothing can be considered definitely established. We implicitly test for monocular stereopsis weakness in the course of this experiment. We also have no data on the dominant eyes of our participants, so we include this as an implicit factor of uncertainty in our data.

## Experiments

As reported earlier, we conducted two mutually complementary experiments, denoted I and II. In the final analysis, we also freely draw on the extensive material collected in our previous study (Koenderink et al., 2004). The difference is the emphasis on the phenomenology of the matter. Spookiness cannot be investigated with purely objective methods; it has to do with how things appear. We address aspects of visual awareness, rather than physiology or proper psychophysics.

Half of the observers (randomly selected) started with Experiment I and the other half with Experiment II, this in order to avoid sequence effects.

In the analysis, we prefer to “let the data speak for itself,” that is to say, we practice explorative data analysis. We freely use Bayes factors instead of *p* values where this appears indicated (Jaynes, 2003; Jeffreys, 1961). In the discussion sessions, we cautiously interpret these data.

### Experiment I

In Experiment I, we attempted to probe the introspective reports of the observers and investigate whether there might be anything resembling a unique “majority report.” Such would strengthen the value of singular first person reports due to the power of intersubjectivity. In this experiment, we explore simple questions that are close to criteria as used in regular psychophysics.

Since many participants are not familiar with various formal concepts from geometry, we started by showing examples of rigidity, non-rigidity, and partial rigidity. The examples were short video clips (provided on the publisher's website) showing abstract 3D objects transforming in various ways, they did not involve pictorial elements used in the actual experiment.

An example is (absolute or relative) “rigidity.” By varying a parameter in a dynamic stimulus, one might indeed determine an objective threshold for noticing “rigidity.” One might even do this without a notion of rigidity as a quality of visual awareness. Asking for “rigidity” is asking whether a certain quality is present in visual awareness. One addresses a quality that purports to pertain to what is experienced as “external visual object.” Yet, it is not clear that participants fully externalize such an experience (see later). Some may rely primarily on emotive feelings of various kinds.

### Methods of Experiment I

**Figure 2.**
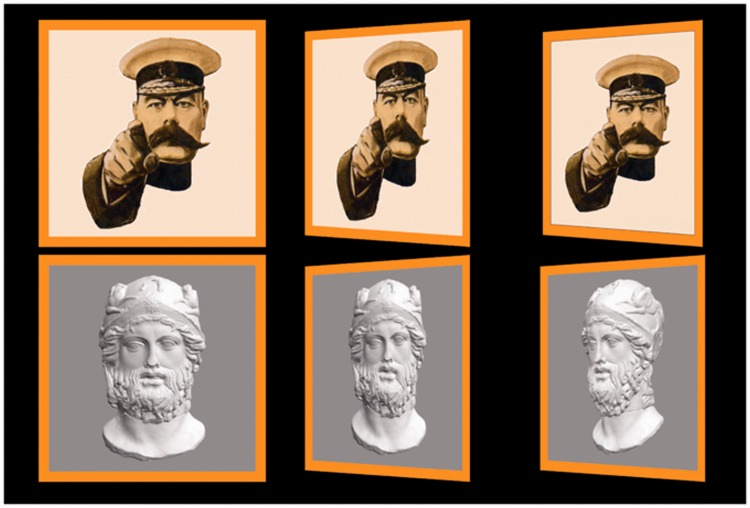
The top row shows the cases involving the Leete poster and the bottom row those involving the Athenian warrior. The left column contains the frontal presentations. Top-center is a frame from the video clip C1, top-right from the video clip C2. This is far more striking in motion: in Case C1, the portrait rotates with the frame and in Case C2, it is motionless (always frontal). Bottom-center is a frame from the video clip C3, bottom-right from the video clip C4. This is again far more striking in motion: in Case C3, the portrait is flat and in Case C4, it is volumetric (in both cases it rotates with the frame).

**Figure 3.**
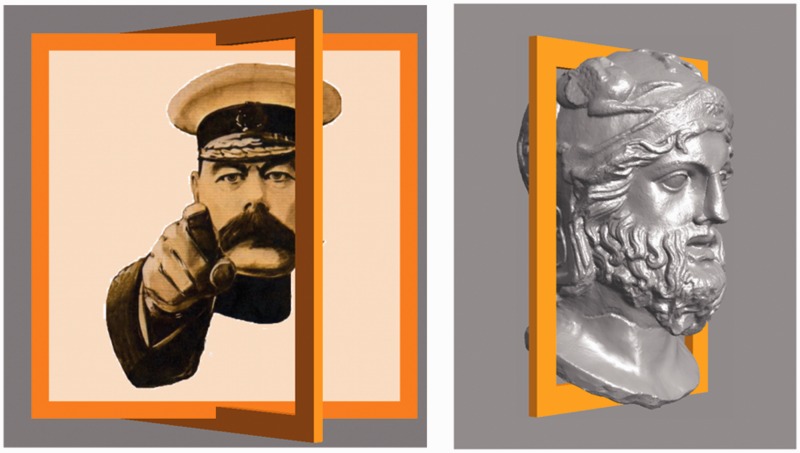
Such extreme poses as shown here were not actually used in the experiment, but they perhaps serve better than Figure 3 to illustrate what these cases attempt to target. In the case of the Leete poster (left, Case C2), the frame rotates whereas the portrait stays frontoparallel. The picture plane defined by the frame does not coincide with the “actual” picture plane. In the case of the Athenian warrior (right, Case C4), the frame and the head rotate rigidly together, but the head sticks out from the nominal picture plane as defined by the frame. In the poses depicted here, occlusions give away the actual spatial relations. In both cases, the “frontal” case is, in principle, not different from the leftmost column of Picture 2. We have slightly adjusted the frame dimensions here to make sure the depth structure is as explicit as can be (in the actually stimuli there were no such telling occlusions). In our experience, many naïve observers fail to fully grasp these relations when occlusions do not “give them away” in the bluntest manner.

Stimuli in the experiment are four short (3-s each) video clips, three of these apparently involving portraits, that are flat counterfeits of some person's face. In one case, the graphics was actually that of a rotating solid head, rather than a flat picture. The clips show continuously varying views of the portrait, the visual direction varying uniformly between 40∘ away from frontal (0∘) to either side of frontal, an 80∘ sweep. The frame is seen to move between two extreme views, first one way and then back the other way. In one case, only the frame moved, whereas the (flat) portrait remained stationary. See Figure 2 for the actual stimuli. Figure 3 shows extreme versions, which may serve to understand the design of the stimuli better.

These are summary descriptions of the four cases:

*Case C1* depicts the Leete poster, framed and with lettering removed. The frame is a rectangular, flat, orange, ribbon, coplanar with the picture panel. Depiction is in proper perspective for the vantage point of the observer (the midpoint of the interocular segment). See Figure 2 (top center).
*Case C2* again depicts the Leete poster, but it is always depicted as seen frontally, whereas only the frame moves. Thus the plane of the frame only momentarily coincides with that of the picture panel, namely when depicted in frontal view. See Figure 2 (top right) and Figure 3 (left).
*Case C3* depicts a portrait of an Athenian warrior (Wenman, 2015), seen frontally. This video clip is just like that of Case C1 (the rotating Leete poster) except for the fact that another person (or rather object) is depicted. See Figure 2 (bottom center).
*Case C4* again depicts the portrait of the Athenian warrior, as in Case C3. However, here the depiction is the rendering of a 3D, volumetric object. Thus the face does not look planar when the view changes, but looks articulated in three dimensions (at least, to many observers). The frame is flat, its plane passing through the center of the 3D head. See Figure 2 (bottom right) and Figure 3 (right).

Notice that Cases C1 and C3 are at least conceptually *identical*: an *en face* portrait rocking back and forth about a vertical axis. The only reason to admit this replication is that we cannot implement Case C4 for the Leete poster. The idea is that Cases C3 and C4 and C1 and C2 yield the interesting comparisons. Thus, all interesting comparisons are limited to C1 and C2 and C3 and C4, other pairings making little sense, except for the trivial comparison of C1 and C3 where we expect similar responses.

There are five questions to each Case C1 to C4, each to be answered with *yes, hmm*, or *no*, the same five questions for each clip. This is different from the conventional forced choice method (*yes* or *no*). The notion is that in 2afc methods participants are forced to select an alternative, even if neither alternative makes sense to them. This is indeed exactly what one wants from an objective method, for ideally neither the observer nor the person running the experiment should have the slightest notion of what is going on. “Double blind” is desirable when objectivity is what one strives for. This makes sense in objective studies because (subjective!) “meaning” should not play any role.

In contradistinction, in experimental phenomenology, the *subjective meaning* of the alternatives is crucial. Thus, the participant should be able to indicate that *neither alternative makes sense*. This implicates that the alternatives *yes, hmm*, and *no* are not members of a common continuum nor members on a common “three-point scale.” *Hmm* is categorically *external* to the range spanned by *yes* and *no*. Consequently, in our analysis, we will treat the task in terms of two distinct binary choices: “does the question make sense” and if so, “is the preferred alternative *yes* or *no*.” This has consequences for the formal analysis.

The questions are:

Q1: does the frame appear to rotate?

Q2: does the frame appear to deform?

Q3: does the face appear to rotate?

Q4: does the face appear to deform?

Q5: does the face rotate with the frame?

Question 1 and 3 address rigid motion (Euclidean rotation) and Question 5 *relative* rigid motion. Questions 2 and 4 explicitly address the experience of *deforming* shapes.

Questions 1 and 2 relate to the frame, which was presented identically in all four cases. Questions 3 and 4 relate to the face. Question 5 relates to the face in relation to the frame. Thus, it makes sense to consider the pair Questions 1 and 2 and the triple Questions 3, 4, and 5 in the analysis.

In the analysis, we refer to the presentations as “Case 1…4” (or just “C1…4”) and to the questions as “Question 1…5” (or just “Q1…5”), sometimes paired as for instance “C2–Q3.”

The participants view a clip, then the display changes to the list of questions with three possible responses for each of the fve questions, each choice indicated as a button. The participant clicks (using the mouse) on a button. Once clicked, the three buttons for that particular question become unavailable. When all five questions have been responded to, the program proceeds.

### Results of Experiment I

The possible responses are *no, hmm*, and *yes*. We have 4 (Cases) × 5 (Questions) is 20 answers per observer. We look for recurring patterns (over observers) in the responses.

We start by discussing the results of Group A, which is composed of naïve observers and thus likely to have the most diversity. It is also the largest group and thus will yield the strongest statistics. We discuss Groups B and C in relation to A, thus more summarily, mainly looking for notable differences.

First thing to do with a large group of naïve participants is to screen observers, in this case to check for consistent formats of the reports. We had to remove a few participants because one or more answers were missing. The group we report on in this section includes 131 observers.

As a first pass through the data, we collect instances of *hmm* responses. In total, there are 57 of those, implying a frequency of 2.2% (credibility range 1.7–2.8%), which is rather low. There are eight observers that responded *hmm* four times, which seems excessive. Indeed, a Poisson dispersion test shows that the number of *hmm* responses per observer is very far from truly random. The tendency to use *hmm* is highly idiosyncratic, as is only to be expected.

**Figure 4.**
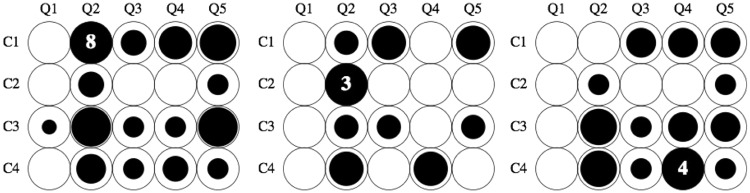
The distribution of *hmm* decisions for Groups A, B, and C (left to right) over all cases and questions. The total number *hmm* decisions is rather low, we have indicated the number of instances for each group. Notice that the pattern of responses is similar for all groups, with the notable exception of *hmm* decision for C1–Q2 in Group C (“does the frame deform?” for the generic Leete poster case). The area of the black disks is proportional with the frequency.

Of course, it is of interest to see how the *hmm* responses are divided among the cases and questions. This distribution is far from uniform as can be gleaned from Figure 4.

A comparison with the results for Groups B and C (Figure 4) suggests that the pattern is systematic, although there also appear to be inter-group differences. The main difference is C1–Q2. Group C never hesitates whether the frame deforms, Group B does and for Group A, it is the major source of *hmm* decisions. Perhaps remarkably, this does not repeat in C3–Q2.

Fact I.1: A minority of observers find it hard to decide whether the frame deforms or not.

After considering the *hmm* responses, we concentrate on the *yes–no* answers. In doing so, we omit the instances of observers using *hmm*. Although this is perhaps excessively conservative, we have sufficient data not to worry about it. We specifically look for patterns in the data that might fit the intention in setting up the questions. To make sure no important regularities are willy nilly ignored, we also performed various types of analysis that are not reported here. This makes that we are quite certain to report on the major features here. We discuss these by groups of cases and questions.

### All Cases, Questions 1 and 2

The Questions 1 and 2 address the mode of change of the frame, thus it appears natural to look over all cases. After removal of the *hmm* responses, we end up with 501 *yes–no* responses. There are 23 *hmm* responses, 22 of which are of the Type YH (we use this notation as shorthand for “first question *yes*, second question *hmm*”). Thus, these observers experience the frame as rotating, but are not fully decided on whether it deforms. This corresponds to the aforementioned Fact I.1.

Notice that the four possible response patterns are nn, yn, ny, and yy. In case there was no regularity, these would be equally likely, thus drawn from a binomial distribution with probability 1/4. If there is a discernible pattern, it should deviate from this in a remarkable way. To quantify this, we compute the Bayes factors.

The Bayes factors are reported in bits. As a metric, we use the scale:

**< 1 bit**: ***negative***

1–2 bit: *anecdotal*

2–6 bit: *positive*

6–10bit: *strong*

> 10bit: *compelling*

Intuitively, a Bayes factor of 10 bits implies that the hypothesis that *the pattern exists* predicts the observation a thousand times (210=1024) better than the non-informative null hypothesis does. Thus, the Bayes factor is *evidence in favor* of the existence of the pattern. The logarithmic measure makes the evidence an additive quantity. The majority of conclusions reported here are in the “compelling” category, except for those that apply to the smaller groups, in such cases, we explicitly mention the strength of the evidence.

**Figure 5.**
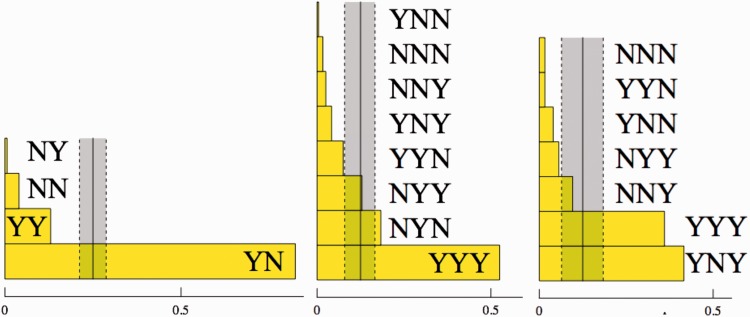
Left: The frequency of occurrence of a pattern (scale [0,1]) for the pair of questions Q1,2 and all cases for Group A. “*Does the frame rotate?*” and “*Does the frame deform?*”. The Pattern YN is much more frequent than the binomial null hypothesis predicts. The latter prediction is the black vertical line, and the gray band gives the two-sided 5% confidence interval. The Patterns YY, NN, and NY occur much less frequently than predicted by the null hypothesis. The Bayes factor > 10 bit tells one that the existence of the Pattern YN is “compelling.” Thus, the pattern is that over all cases “*The frame does appear to rotate rigidly*” (Pattern 1). Center: The frequency of occurrence (for Group A) of patterns involving Q3, 4, 5: “*Does the face rotate?*”, “*Does the face appear to deform?*”, and “*Does the face appear to rotate with the frame?*” (we already know that the frame almost always appears to rotate). This involves both generic cases (C1,3). There is “compelling” evidence in favor of the Pattern YYY: “*The face appears to rotate with the frame AND it appears to deform*” (Pattern 2). Right: The frequency of occurrence (for Group A) of patterns involving Q3, 4, 5: “*Does the face rotate?*”, “*Does the face appear to deform?*”, and “*Does the face appear to rotate with the frame?*” (we already know that the frame always appears to rotate). This involves C4, the 3D rotating Athenian warrior. Here, there is “compelling” evidence in favor of both Pattern YNY: “*The face appears to rotate rigidly with the frame*” and YYY: “*The face appears to rotate with the frame AND the face appears to deform*” (Pattern 4).

The advantage of the Bayes factors is that they fit our explorative endeavor much more naturally than conventional hypothesis testing. It allows one to collect and quantify evidence in favor of a hypothesis instead of against some null hypothesis. To offer some solace to those who mistrust Bayesian methods, we also show the 5% confidence interval for the binomial case, as in Figure 5 (left). In no case do we report patterns that would not lead to very small *p* values in a classical hypothesis test.

In this case, the pattern is very clear, namely a huge majority yn (Bayes factor > 10: “compelling”). We find exactly the same pattern for the other groups. Thus, the evident consensus for all groups is:

**Pattern 1.** For all groups and all presentations the frame appears to rotate rigidly.

This is perhaps not a great surprise (Saunders & Backus, 2007).

#### Cases 1 and 3, Questions 3, 4, and 5

This involves the “standard” stimuli. One expects similar results for these cases. Indeed, they are not different (*p* value .35) in a *t* test, so we pool these cases. Possible patterns are nnn, ynn, … , yyy, eight in total. There is one pattern that dominates; it is yyy (Figure 5, center). For Group B, there is no clear pattern, for Group C there is, again yyy. (In all cases, Bayes factor > 10: “compelling.”) Thus:

**Pattern 2.** For Groups A and C and for the generic stimuli, the face appears to rotate with the frame and the face appears to deform. For Group B, there is no discernible pattern.

#### Case 2, Questions 3, 4, and 5

This involves the special case of the Leete poster in static, frontal position with a rotating frame. Again, there are eight possible patterns. For Group A, there is one dominating pattern, it is nnn. For both Groups B and C, the pattern is also nnn. (In all cases, Bayes factor > 10: “compelling.”) Thus:

**Pattern 3.** For all groups and for the static, frontal presentation with rotating frame, the face does not appear to rotate (unlike the frame) and it does not appear to deform.

#### Case 4, Questions 3, 4, and 5

This involves the special case of the 3D model of the Athenian warrior that rotates with the frame. Again, there are eight possible patterns. For Group A, there are two dominating patterns, they are yny and yyy (Figure 5, right). As in all cases for Group A, the evidence is rated “compelling.”

For Group B, we find only the Pattern yny, although the Bayes factor is only 3.9 bits (“positive”). For Group C, we find the Pattern yny (Bayes factor 7.9 bits: “strong”), as well as the Pattern yyy (Bayes factor 4.6 bits: “positive”). Thus:

**Pattern 4.** For all groups and for the 3D, rotating Athenian warrior, the face rotates with the frame. Groups A and C report both deformation and rigidity and Group B only rigidity.

**Figure 6.**
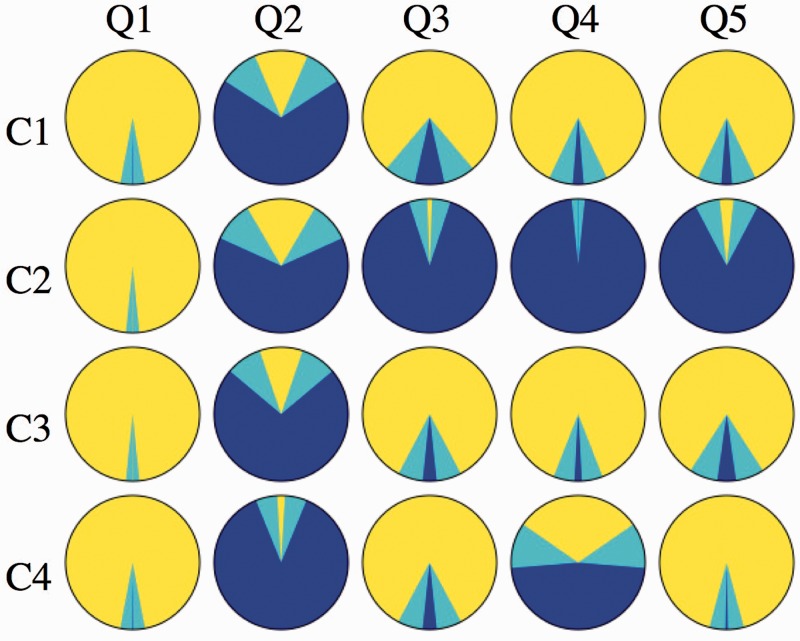
The overall pattern of responses for Group A. The *yes* counts are indicated by yellow and the *no* counts by blue. The cyan bands indicate the credibility intervals based on a 5% credibility level. Probability is calculated by Bayesian estimation, using the binomial distribution and the non-informative Jeffreys prior.

Similar results are seen in simple frequency counts over observers (Figure 6). We define the group response as the probability of *yes* among the *yes*–or–*no* answers. This probability is calculated by Bayesian estimation, using the binomial distribution and the non-informative Jeffrey's prior. The credible intervals are based on a 5% credibility level.

Although the resulting distribution warrants close study, it would appear that the search for patterns as described earlier has largely exhausted the structure. To look “deeper” into the data, one might attempt some form of “blind source separation.” Such an analysis might be able to resolve “the voices by which the data speak.”

**Figure 7.**
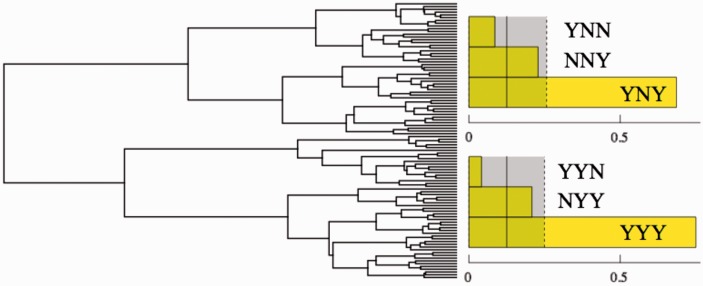
Left: Dendrogram for the responses of participant of Group A in Experiment I. We used Mathematica's DendrogramPlot[] function. Agglomeration for Experiment I was based on the Euclidean distance function and Ward linkage on the normalized responses per observer. It may be regarded a form of “blind source separation.” The major groups are evident, they occur after the first “split.” Right: Like Figure 5 (right) but the analysis performed on the main two clusters of Group A. Notice that the division in clusters resolves the ambiguity in the overall group response. One cluster has the YNY pattern, the other cluster the YYY pattern.

A simple way to perform such an analysis is by agglomeration. We first render the answers numerically (*yes* →+1, *hmm* →0, *no* →-1), thus obtaining 131 vectors in a 20D space. We normalize, that is to say, each vector is transformed to zero mean unit variance. Conventional linear methods such as principal components analysis find not much structure in this data, since it requires 10 dimensions to explain three quarters of the variance. We then agglomerate, using Ward linkage and Euclidean distance, leading to the dendrogram shown in Figure 7 (left).

Apparently, there are at least four major clusters, all of appreciable size (45 in Cluster 1, 39 in Cluster 2, 20 in Cluster 3, and 17 in Cluster 4). Now, we can limit the search for patterns to single clusters.

This does indeed allow one to resolve the ambiguity left in Pattern 4: Cluster 1 has yny and Cluster 2 has yyy as the dominant pattern (Figure 7, right).

**Fact I.2**: For Group A and for the 3D, rotating Athenian warrior, the face rotates with the frame. There is a cluster of observers reporting deformation and another cluster reporting rigidity.

Likewise, we further refine Pattern 2. Clusters 1, 2, and 4 yield the same pattern as the group responses yyy, but for Cluster 3, the pattern is nyn (Bayes factor > 10: “compelling”). Thus:

**Fact I.3**: *There is a cluster for which the generic presentations are experienced differently: The face does not appear to rotate, but it deforms*.

For Groups B and C, clustering does not make much sense (and was not attempted), simply because the group sizes are too small.

### Discussion Experiment I

What do these results imply? The overall answers of the Group A of naïve observers yield a very clear picture, which needs only be nuanced slightly when Groups B and C are taken into account.

The participants happen to report impressions that closely resemble the awareness of the authors when viewing the video clips. The reader is invited to watch these clips on the publisher's website, for an understanding of our conclusions will be virtually impossible without such a personal experience. In various cases, it may be necessary to view a clip several times, with different questions in mind.

There is no doubt as to the efficacy of the video presentation—which, after all, takes place on a flat screen: To the question “does the frame rotate?” 95.4% of the answers were *yes* and to the question “does the frame deform?” 82.8 responses were *no*. Apparently, most observers experience a *dynamic scene in cinematic space*. However, in view of Fact I.1, the decision of whether the frame rotates *rigidly* appears to be not immediately obvious.

Surely, Pattern 1 is a decisive result: *the frame rotates rigidly*. This is important in attempting to make sense of the patterns that apply to the faces. We start with Pattern 2, which applies to the generic stimuli. Observers apparently experience the Leete poster and the flat, frontal portrait of the Athenian warrior, both rotating in a frame as *similar*. The faces appear to rotate with the frame, as is indeed what the graphics simulates. However, the faces are simultaneously seen to deform, how can that be? Notice that (Fact I.3) there is a cluster for which the face does not rotate at all, but only deforms. Are these findings in conflict?

In our experience, one may indeed be aware of *either* rigid rotation with the frame in cinematic space *or* of a planar deformation (periodic shrinking and expanding in the horizontal direction) in the plane of the display screen. The latter mode appears mainly when the frame is not too oblique, the former when it is near its most extreme slanted positions. It seems likely that the observers tried to somehow force such (not necessarily present in their discursive mind) complex visual awareness into the straitjacket of our questions.

The special cases do not yield major surprises. In the case of the static, frontal Leete poster, observers experience the situation much as the graphics simulates (Pattern 3). This is useful as a reference, perhaps a “sanity check,” but it hardly suggests a novel conceptual perspective. It serves to rule out some (in our view far-fetched) interpretations though, we do not explicitly discuss these.

In the case of the 3D rotating Athenian warrior, most observers also experience exactly what the graphics simulates; however, a minority sees the face deform (Pattern 4 and Fact I.2). What to make of that? It is potentially interesting, for apparently, some observers relate the (momentary!) picture of the face to the frame, whereas others do not. If they do, then there is obvious (at one or other pictorial level) deformation, otherwise there is none (for all is accounted for!).

### Experiment II

In Experiment II, we use methods of “fit” to sample the apparent spatial attitude of the brick wall and the frame and methods of “reproduction” to sample the apparent direction of gaze and the apparent aspect ratio. We have explored the method of fit in many previous studies (starting with Koenderink et al., 1992). Both methods of reproduction are novel and were designed for the present experiment.

All methods were experienced as “natural” by the participants. They are of a primary visual nature and involve no, or hardly any, reflective thought. Indeed, it is hard to imagine how reflective thought might aid in performing (“solving” does not really apply here) the task. The settings simply have to “look good” and “feel good.” Again, this is experimental phenomenology, rather than cognitive science.

### Methods of Experiment II

**Figure 8.**
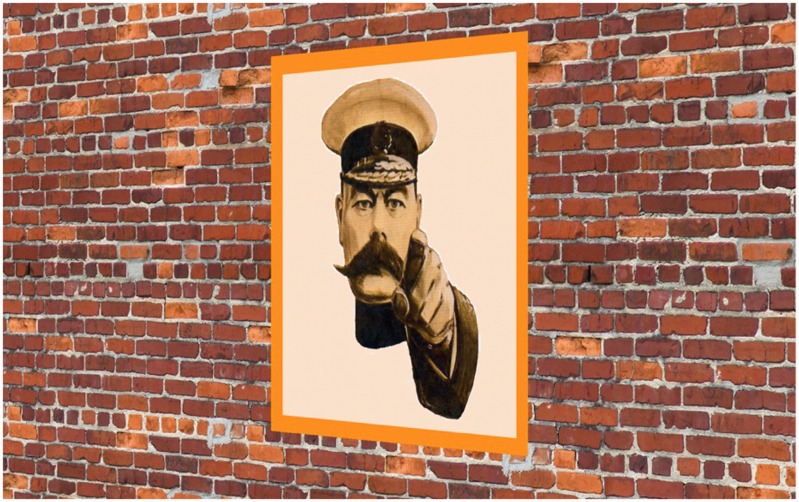
The presentation of the brick wall filled the full window.

Since the stimuli in Experiment II are static, we use the brick wall background (Figure 8) to enhance the awareness of slant of the picture panel. The brick wall was not used in the video clips of Experiment I because the dynamic presentation already yields a vivid impression of spatial attitude.

**Figure 9.**
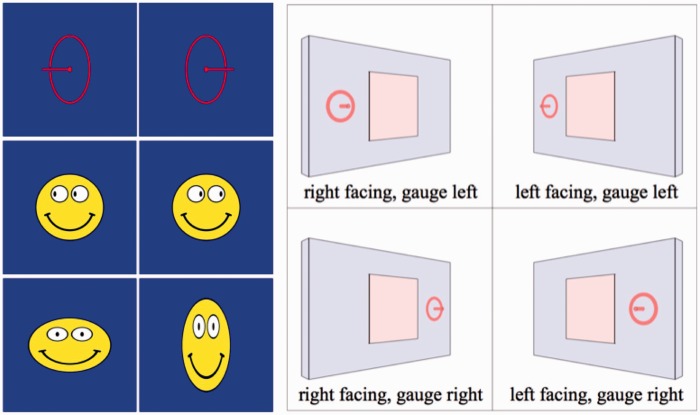
This figure introduces the gauge objects and some technical terms used to describe them in the text. The gauge object for slant is illustrated at left–top, the gauge object for gaze direction at left–center, and the gauge object for facial shape (aspect ratio) at left–bottom (notice that a single graphical object is used to gauge gaze and shape. However, its meaning and user interaction interface are very different in the two cases). The gauge object for slant is the graphical rendering of a circle, indicating a flat disk and thus a planar spatial attitude and a rod sticking out at right angles from the plane of the disk. The combination of disk and rod enables good discrimination at all spatial attitudes. The rod is supposed to stick out forwards (toward the observer) in all cases, so it resolves the mirror ambiguity about the fronto-parallel plane. The example at left has negative slant and the one at right has positive slant. The gauge object for gaze direction is a smiley icon. The position of the pupils in the eyes yield a sensitive measure of gaze. The example at left has negative gaze angle and the one at right has positive gaze angle. The gauge object for shape is also a smiley icon. The example at left has aspect ratio greater than one (frog-faced) and the one at right has aspect ratio smaller than one (horse-faced). At right is shown the geometrical convention referred to in the text. The graphics simulates a wall that is either “left facing” or “right facing.” Gauge objects are placed alongside the stimulus (central square), either on the left or on the right. This makes a difference, as explained in Figure 10 (for a “right faced” attitude, but the alternative is related by mirror symmetry about the vertical).

In one setting, we have the participant indicate slant by fitting a simple elliptical gauge figure to the surface of the wall (Figure 9, left–top; Koenderink et al., 1992). The ellipse should look like it is a circle painted on the wall. The participant controls the simulated slant by means of the left–right arrow keys on the keyboard.

In a second setting, we have the participant indicate the gaze direction of the portrayed head by reproducing that gaze in the picture of a simple smiley (Figure 9, left–center). The participant controls the simulated gaze (in the smiley picture) by means of the left–right arrow keys on the keyboard. (The gaze direction was simulated by the position of the pupil in the smiley's eye. The offset was taken proportional to the sine of the simulated gaze angle.) As one changes the gaze parameter, one experiences the smiley to change viewing direction in a very lifelike manner, despite the fact that the smiley looks like a flat, icon-like picture at all times.

In a third setting, we have the participant indicate the shape of the portrayed head by approximating that shape in the picture of a simple smiley (Figure 9, left–bottom). The participant controls the aspect ratio (width over height) of the smiley by means of the up–down arrow keys on the keyboard. As one changes the aspect ratio, one experiences the smiley to change from “frog faced” to “horse faced” (or *anuran* to *equine* if you like). The setting feels like a natural one.

Three slants are presented, frontal and 40∘ either side from frontal. The gauge figures are presented once at the left of the frame and once at the right. Thus, the participant has to complete 18 settings in total. All conditions are fully randomized.

In Figure 9 (right), we illustrate the cases simulated by the graphics. The wall may be frontal or oblique. If oblique, we distinguish two different attitudes, referred to as the “left facing” and the “right facing” wall. In all these cases, the gauge objects may appear either to the left or to the right of the stimulus. For most of the discussion, we present averages over the latter two cases, but in a few instances we differentiate. Notice that Figure 9 (right) was drawn for clarity and is only intended as schematic. In reality, the screen of the display unit was a fixed rectangle in any case.

### Results of Experiment II

Again, the first thing to do is to screen observers. We use three criteria to omit observers from the analysis:

1. If one or more slant settings are below the 2.5% or above the 97.5% quantiles, we omit the participant;
2. If one or more gaze settings are below the 2.5% or above the 97.5% quantiles, we omit the participant;
3. If one or more aspect ratio change values is higher than 1.2, we omit the participant.

This reduces the number of observers in Group A from 166 to 131.

*As to Criterion 1*, this simply removes a few observers that most likely did not understand the task, did something random, or simply did nothing at all (left it at the random initial setting);

*As to Criterion 2*, a few settings are to the extreme left or right (almost 90 ° gaze angle), these are evidently erroneous. Leaving them out seems natural.

*As to Criterion 3*, some participants appear to see smiley faces become two or more times wider when viewed obliquely than when viewed frontally. What happens, as suggested by personal interactions, is that these observers interchanged the horizontal and vertical dimensions. Indeed, if one swaps these, their results become generic. In view of the large enough numbers, it seemed best to omit such observers.

The selection process was carried out fully automatically by an algorithm based on these criteria without exception.

Thus, we were left with 131 participants in Group A. Likewise, we retained 7 of 13 participants in Group B and 20 of 25 participants in Group C.

**Figure 10.**
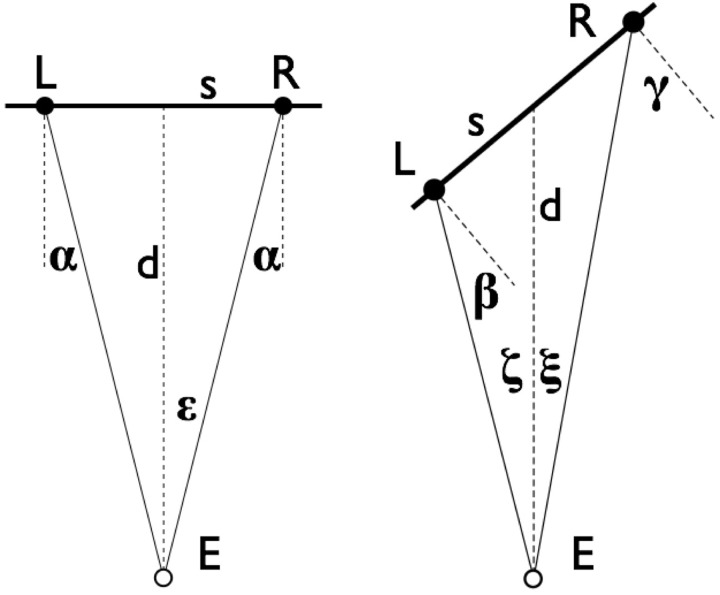
The geometry of the stimulus. These schematic drawings are in the space that is simulated by the graphics (the display screen is frontoparallel in all cases!). In this drawing, E denotes the “eye” (actually the observer's mid-ocular-segment or dominant eye), d the viewing distance (57 cm), and s the wall simulated by the graphics (15 inch horizontal extent). In the drawing at left (frontal view), the locations L and R are places where the smiley gauge might occur. In the drawing at right, L and R denote the places where the slant gauge figure might occur. The eccentricities of L and R (angles *ε, ζ* and *ξ*) are about 15∘ of visual angle. The smiley subtends about 8∘ the slant gauge figure about 5∘. Of special interest are the angles *α, β*, and *γ*. The angle *α* is 14.2∘, *β* is 25.6∘, and *γ* is 50.2∘. The slant of the wall is 40∘.

The geometry of the stimulus is worth special attention (Figure 10). Of course, this geometry is partly defined by the *physical setting* (display unit, participant location), partly by the *pictorial content* (the perspective rendering of the brick wall, the frame, etc.). The effect of the geometry depends on the way the participant experiences the pictorial space. This is an idiosyncratic factor that we cannot control. However, we have considerable experience with such effects for the generic population from our earlier work (Koenderink et al., 2001; Koenderink & van Doorn, 1995, 2003, 2012; Koenderink et al., 2011; Koenderink et al., 2009). Figure 10 is a drawing in the space simulated by the graphics, thus it is observer independent.

The actual situation is even more complicated, since the observers view the screen *binocularly*. Their responses are likely to be mainly determined by the location of their dominant eye or perhaps by the location of the virtual “cyclopean eye” in case eye dominance is weak or absent (Chaurasia & Mathur, 1976; Khan & Crawford, 2001; Porac & Coren, 1975). Since we have collected no eye dominance data, this implies a factor of uncertainty in the observations.

The stimulus proper is always presented at the center of the screen. The area around it is filled with the brick wall. The wall texture is very important in pictorially specifying the slant of the frame. The smiley gauge is presented either on the left or the right of the stimulus (Figure 9, right). In the final analysis, we average over the two cases, but it is of some interest to consider the relevance of these placements. We have shown previously that generic observers relate the spatial attitude of an object to the visual direction to that object (Koenderink et al., 2009). This suggests that the participant would experience the smiley as slanted over an angle α (about 14.2∘) in the “frontal” view. However, this effect of “external local sign” (Koenderink et al., 2010) is especially important if the object is volumetric. Since the smiley is flat and not seen as “on the wall,” observers may well experience it as normal to the local viewing direction and thus act as if *α* were zero. This is an issue that has to be resolved empirically, we address it in our analysis.

The gauge figures for slant are also presented once to the left and once to the right of the stimulus, conditions to be averaged over in the final analysis. This case is different from the previous one, for various reasons. Notice that the angles *β* (about 25.6∘) and *γ* (about 50.2∘) are very different. Their mean is about the slant of the wall (40∘), but one angle is much smaller and the other much larger than that. Another reason this case is very different from the previous one is that the observers are expected to “see” the ellipse of the gauge figure as being in the plane of the wall (Koenderink et al., 1992). In our analysis, we need to consider the *four cases* of wall slanted to the left or right and gauge presented to the left or right as essentially distinct. Our analysis fully takes this into account. However, for the sake of conciseness, we present results in terms of suitable averages, occasionally mentioning systematic deviations where relevant.

The gauge figure settings for obliqueness and the gaze settings are converted to degrees of visual angle. The aspect ratio settings are reported as the ratio between the setting in oblique view to that in the frontal view. The latter *normalizes* aspect ratio estimate differences between participants. This is very important because absolute aspect ratio settings are inevitably idiosyncratic. We refer to this ratio as the “aspect ratio change.” It will be equal to one in the case of “no effect.” Any value different from one implies a non-rigid deformation. As discussed here, we find a very significant effect of obliquity in that the face appears to grow leaner as the slant increases.

In the frontal view, the spatial attitude of the frame and the direction of gaze coincide. Both the normal to the frame and the gaze direction point straight toward the observer.

In the oblique presentations, the spatial attitude of the frame is indeed indicated as oblique. There is a clear dichotomy between a slant to the left and one to the right. Such a dichotomy is much less or absent for the gaze direction, which remains largely unaffected by the oblique presentation. This is, of course, an objective demonstration of the familiar effect that frontally counterfeited people always “look at you” or “follow you” when you walk past the portrait—that is to say, the “uncanny effect.”

We start the analysis with Group A, the large group of 131 naïve observers.

**Figure 11.**
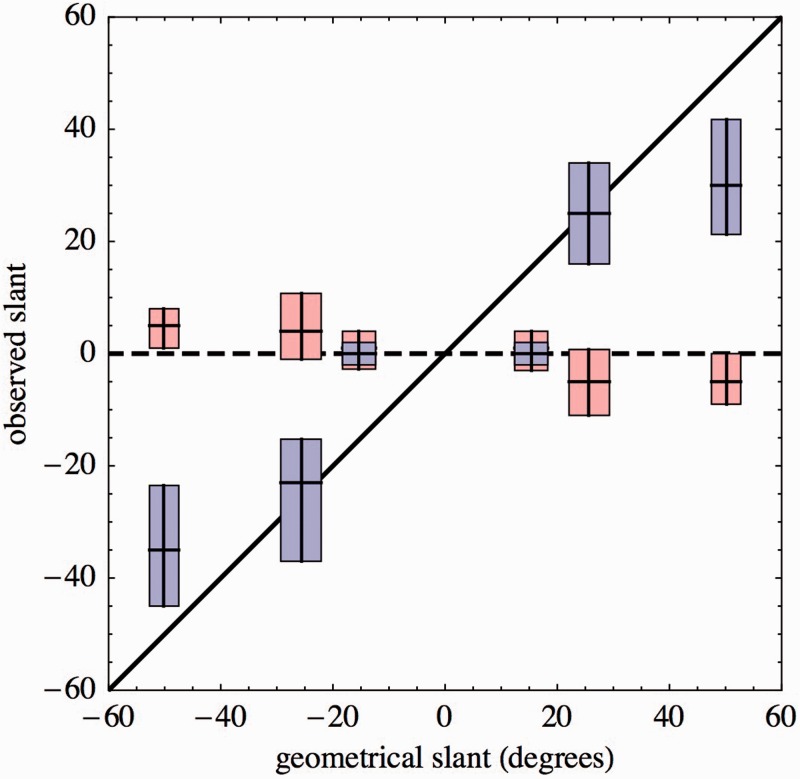
An overview of the slant (blue) and gaze (red) settings for Group A. The top and bottom of the boxes, with the horizontal line roughly at center show the quartiles of the settings. The vertical centered line shows the angles as obtained from Figure 10. These “geometrical angles are the angles ±α,±β, and ±γ.” The left and right sides of the boxes show the bounds on the variation of these angles as the (unknown) eye dominance is taken into account. The black diagonal indicates the identity (settings equal to the angles simulated by the graphics) and the dashed horizontal line indicates independence (the settings are frontal no matter what the graphics is like).

**Figure 12.**
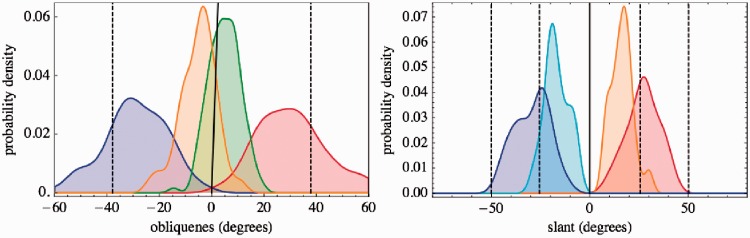
*Left*: smooth histograms of the slant and gaze settings of Group A observers for the two oblique spatial attitudes simulated by the graphics. The vertical dashed lines show the predictions from the geometry explained in Figure 10. In order not to complicate the figure too much, we averaged over the positions of the gauge objects. The slant settings for right facing walls are in red, the gaze settings in orange, whereas the slant settings for left facing walls are in blue, the gaze settings in green. *Right*: Here, we show the slant settings for left and right facing walls, but differentiated with respect to the location (left or right of the stimulus pane) of the gauge object. (These are data from Group C, selected because the data of the group of these (many professional) observers has less variability than Group A.) Notice that the observers clearly resolve the geometry explained in Figure 10, although the slant as simulated by the graphics is severely underestimated. The same differentiation is found in Groups A and B, although somewhat less articulate.

**Figure 13.**
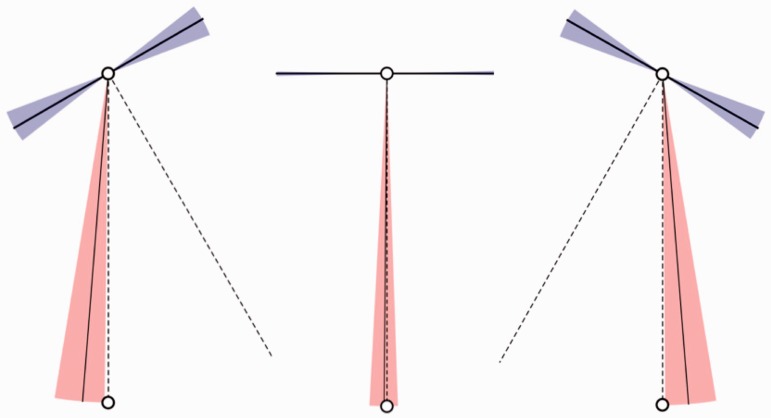
The observations of slant (in blue) and gaze (in red) indicated by their interquartile ranges, plotted in the space as simulated by the graphics. The oblique drawn lines indicate the medians. The dashed lines are perpendicular to the median slant directions. The vertical dashed lines indicate the forward direction. Notice that the gaze is much closer to this forward direction than to the direction perpendicular to the slant. The latter might be seen as the (naïve) prediction of where “the face looks at.”

In Figure 11, we plot the observed slant and gaze angles against those implemented by the graphics. Essentially, the same data is plotted in Figures 12 and 13, perhaps these are even more intuitive. This already reveals the major effects in a nutshell:

**Fact II.1**: *The slant settings for Group A are monotonically related to the angles implemented by the graphics. However, the slant settings for the frontal presentation are “frontal” (irrespective the angle α) and those for the highest slants are severely underestimated*.

**Fact II.2**: *The gaze settings for Group A are unrelated to the magnitude of the angles implemented by the graphics. However, there is a systematic offset from frontal in the opposite direction of the slant. The settings in frontal presentation are “frontal” (irrespective the angle α)*.

The mean (over the gauge object locations) gaze settings for the left and right facing walls are correlated (Pearson correlation coefficient–.25) at the 0.004 level. The gaze settings for a right facing wall and left and right gauge object locations are also correlated (coefficient + .27, at the 10-5 level), similar for the left facing wall.

For the frontal presentation, the gaze settings for left and right gauge object locations are correlated (coefficient + .29 at the 0.0003 level). The gaze directions differ by 1.3∘. If the gauge object is located on the right of the stimulus, then the gaze direction is farther to the left. Similarly, if the gauge object is located on the left of the stimulus, then the gaze direction is farther to the right.

The average gaze directions are -4.9∘ for the left facing wall, -1.2∘ for the frontal wall, and 5.1∘ for the right facing wall. The quartile ranges (-9.4∘ to +0.5∘ for the left facing wall and 0.63∘ to 9.4∘ for the right facing wall) do not overlap. Yet, the distributions are broad enough that the odds are that for a given instance four out of five gazes go to one side and one out of five to the other.

**Fact II.3:** Gaze settings for left and right facing wall attitudes are correlated.

**Fact II.4:** Gaze settings for left and right locations of the gauge object are correlated for the slanted as well as for the frontal presentations.

**Fact II.5:** Gaze direction depends only upon the orientation of the wall, not on the location of the gauge object. Wall facing left implies gaze toward the right and vice versa. For the frontal wall, the gaze is frontal.

Another essential issue involves the aspect ratio change. The median aspect ratio in the frontal presentations is 0.81 and quartile range is 0.69 to 0.93. Thus, most observers indeed attempt to estimate the shape of the head (which is evidently elongated vertically, see Figure 2), they do not simply set a default (circular) smiley. The settings for the smiley on the left and on the right are correlated (*p* value 210-7). The inter-observer variation explains 16.3% of the variance, thus the settings are to some extent idiosyncratic. This is even more pronounced in the other groups. For instance, for Group C (the largest small group), 64% of the variance is explained by the inter-observer variations.

**Figure 14.**
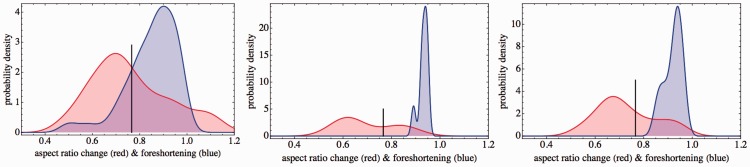
Left: Smooth histograms for the aspect ratio change (red) and the foreshortening factor predicted by the observed slants for Group A; Center: idem for Group B; Right: idem for Group C. The black line indicates the foreshortening factor predicted by the graphics. There is no a priori reason to expect these very different observations to be related except for the simplest geometrical model. Here, it is visually evident that the slant settings are useless to predict the aspect ratio settings. The slant simulated by the graphics comes somewhat closer, but, of course, the observers have no immediate access to that.

Of course, we are mainly interested in the aspect ratio *change*. Here, “change” is used to indicate that the reported values are normalized by the fronto-parallel settings. Ideally, the “aspect ratio change” would be equivalent to the aspect ratio of an object that appears like a circular disk in frontal view. In a naïve model, the aspect ratio change would be due to geometrical foreshortening, the cosine of the angle of the oblique view. Here, we would not know which angle to take. A “veridical choice” might be the angle simulated by the graphics, but the slant setting might be the causally effective angle in the participant's mind. All we can do is compare observations and expectations. In Figure 14 (left), the aspect ratio change is compared with the foreshortening factor derived from the observed slants. There appears to be little relation. Correlation test also reveals the slant and aspect ratio settings to be independent. The result is even more articulate for Groups B and C. Thus:

**Fact II.6**: *For all groups, there is a pronounced aspect ratio change. The aspect ratio change is stronger than expected from the graphics. It is much stronger than the foreshortening factor derived from the observed slants. Slant and aspect ratio settings are not correlated*.

Some other observations are of interest, especially those that involve the relation between various parameters. Here, we mention a few that apply to Group A. The other groups are often too small (or marginal) to arrive at strong conclusions. However, they yield results that never oppose those of Group A and typically echo them in a (statistically) weak form. In Figure 14 (center and right), we show the aspect ratio changes for these groups.

**Figure 15.**
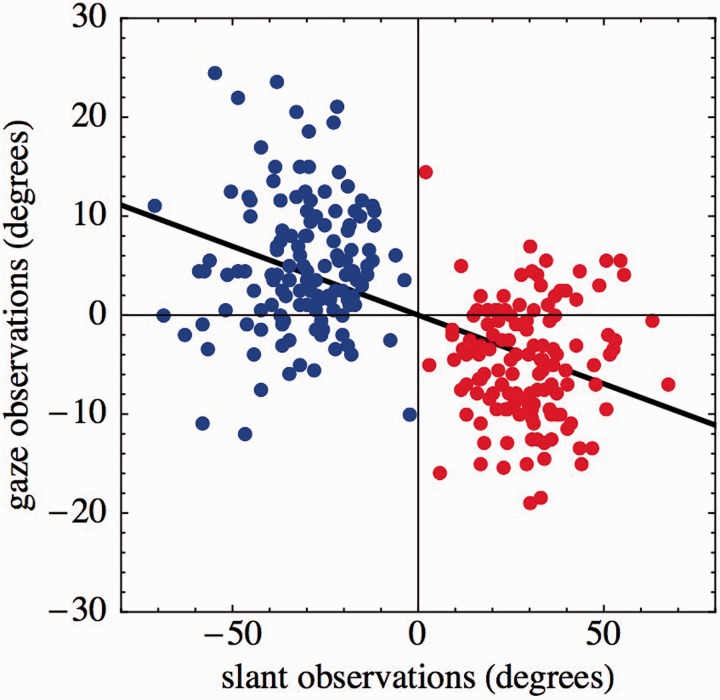
Scatterplot of the slant and gaze of observers in Group A. Notice that the black regression line fits the data, but does not implicate that slant and gaze are related, only that both depend on the direction of the obliqueness simulated by the graphics.

The slant settings for left and right facing walls correlate (*p* value 1.610-6) and so do the gaze settings (*p* value .004); however, the slant settings do not correlate with the gaze settings (*p* values .47–.90). Both the slant and the gaze settings have a left–right dependence, which might suggest a relation (Figure 15), which is obviously spurious.

The gaze settings do not correlate at all with the aspect ratio changes (*p* value .78) nor with the slant settings (*p* value .86).

These are all important observations:

**Fact II.7:** The observations of aspect ratio change have no relation to the observed slants or gazes. They are stronger than expected on basis of the graphics.

**Fact II.8:** The slant and gaze settings are mutually unrelated.

**Fact II.9:** Slant settings for left and right facing walls are highly correlated (so are left and right gaze settings; Fact II.3).

**Fact II.10:** In the frontal presentation, both the slant and gaze settings are frontal, the angle α is not taken into account.

As in Experiment I, one might expect a blind source separation to reveal additional structure. We use data obtained by joining slant, gaze, and aspect ratio change observations. We normalize each of these before joining, obviously necessary because of the categorically different dimensions, then agglomerate using the Euclidean distance function and Ward linking. In this case, we do not find much novel structure, and the major clusters differ mainly in the degree of underestimation of the slant.

Indeed, the slant settings have a very large spread. If we study the slant settings for the case where the simulation from the graphics is 50.2∘, we find a median setting of 33∘ (thus the aforementioned pronounced underestimation), with an interquartile range of 23∘ to 43∘, whereas 10% of the settings are outside the range 11∘ to 56∘. Thus, the variation in the group of 131 participants is more than a factor of 5. The settings for left and right facing walls are correlated (R–squared .16, *p* value 1.610-6).

**Fact II.11**: *Clustering in Group A mainly reveals differences that relate to the slant settings. Whereas one cluster closely follows the expectation from the graphics, another major cluster severely underestimates these. The variability of slant settings is at least a factor of 5. Settings for left and right facing walls are correlated*.

### Discussion Experiment II

All observers experience the gaze direction as aimed at them, albeit with slight variations. Simultaneously, the frame is experienced as changing its spatial attitude over a range of tens of degrees (Facts II.1 and II.2). This evidently illustrates the familiar uncanny effect.

The face clearly has a different shape in the oblique presentations from its appearance in the frontal presentation (Fact II.6). It is as if the expected automatic “correction” (Banks, Rose, Vishwanath, & Girshick, 2005; Busey et al., 1990; Hecht et al., 2014; Yang & Kubovy, 1999) for the perspectival foreshortening is *applied to the frame, but not to the face*. This is a highly remarkable and conceptually important result.

It is of some interest to study how this deformation effect corresponds to the foreshortening cue. Notice that we have six parameters of interest here:

— the depicted slant *S*^*P*^, as given by the perspective;
— the apparent slant *S*^*A*^, as indicated by the participant's settings;
— the slant *S*^*R*^, as derived from the change in aspect ratio;
— the actual foreshortening factor *F*^*P*^, as derived from the depicted slant;
— the foreshortening factor *F*^*A*^, as derived from the apparent slant;
— the deformation *R*, as determined from the observed aspect ratio changes.

The data show that neither *F*^*P*^ nor *F*^*A*^ are well estimated by *R*. Apparently, the aspect ratio changes stand in no relation to either the observed gazes or slants (Fact II.7).

What does this imply? Apparently, the aspect ratio estimates derive from the 2D visual field, whereas the slants must derive from pictorial space, that is to say, monocular stereopsis. This is corroborated by Facts II.7 and II.8, the aspect ratio change, slant, and gaze settings are mutually unrelated.

Such a conclusion fits the finding from Experiment I that the face appears to *deform* despite the fact that it “*rotates with the frame*.” What appears to be the case is that the change of the face over time is experienced as a deformation and a rotation, whereas the frame suffers a rigid rotation. The rotation and deformation of the face can be experienced each as such, simultaneously or successively. It is in such ambiguity of the presentation and logical inconsistency in reflective thought that we have to look for an understanding of the results. Numerous idiosyncratic effects are to be expected.

The two clusters of the Group A observers mainly relate to the strength of the monocular stereopsis as revealed by the magnitude of the observed slants (Fact II.11). Such slant estimate “gain factors” are evidently idiosyncratic as is shown by the correlation between left and right settings (Koenderink & van Doorn, 2003; Koenderink et al., 2001; Fact II.9). This is different from the relation between the gaze direction settings and the slants, which are essentially uncorrelated. It is also different from the relation between the aspect ratio change settings and the slants, which are essentially uncorrelated too.

Gaze direction and aspect ratio change have apparently no relation to monocular stereopsis, whereas the slant observations have. These facts are highly relevant to the interpretation of the main effect.

The gaze directions have an idiosyncratic component, as evident from Fact II.3. There is no dependence upon the location of the gauge object relative to the stimulus (Fact II.5), not even in the case of frontal presentation. Some part of the variance is possibly related to eye dominance, but we have no means to check this. At least one participant spontaneously remarked on the fact that the gauge object is not exactly presented frontally; thus, in order to have the face “look at you,” a small correction in the opposite location might be preferred. Indeed, such an effect might be expected due to the nature of the “external local sign” for the majority of the population, a strong effect that we have investigated before (Koenderink et al., 2009, 2010). However, we find no evidence for such a systematic effect here, possibly due to the fact that the angle *α* is only about 14∘.

**Figure 16.**
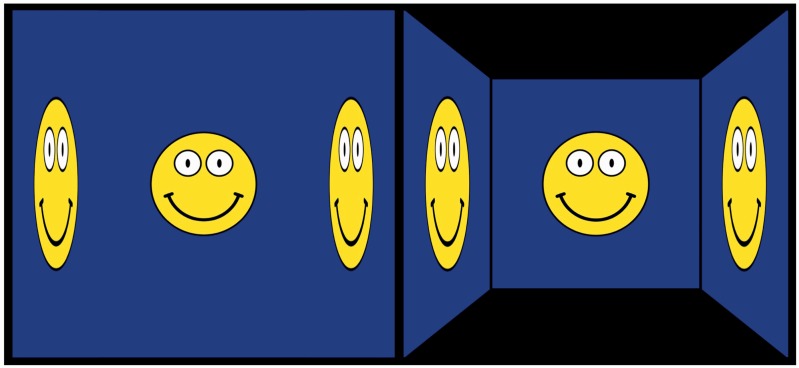
*Left*: A frontal gazing smiley of unit aspect ratio (circular face) and two “horse-faced” smileys, also with frontal gaze direction. This is a purely planar rendering. *Right*: Here, the smileys are identical as at left, but we added a “triptych” in 3D. The smiley at center is drawn on the central, frontoparallel plane, but the horse-faced smileys can be seen in any of two ways: They can be seen as frontal and horse faced, like at left, but they can also be seen as circular, “foreshortened” smileys. The latter happens if you see them as drawn on the oblique side panels—in pictorial space, of course. Consider how you would prefer to change the positions of the pupils in the eyes so as to make the smileys “look at you.” In our case, if the panel faces left, we would set the pupils so as to let the smiley “look (just a little) over our right shoulder” and vice versa. Of course, this supposes that you manage to see the smileys as slanted in 3D in the first place! Apparently, not everybody is able to do that. If you can indeed switch voluntarily between the 2D and 3D awarenesses, you are ready to experiment with the experiences that we believe induced our “average observer” to do what she did.

Lord Kitchener does not “look straight at the observer” (as in frontal presentation: median 0∘) when the wall is oblique, but looks slightly (median 4.5∘) over the right shoulder when the wall is facing left, and vice versa. Notice that this effect is quite appreciable, as it amounts to the interpupillary distance as seen from a range of about 80 cm. It appears that we find quantitative evidence of the “Wollaston illusion” (Wollaston, 1824) here (Figure 16).

**Figure 17.**
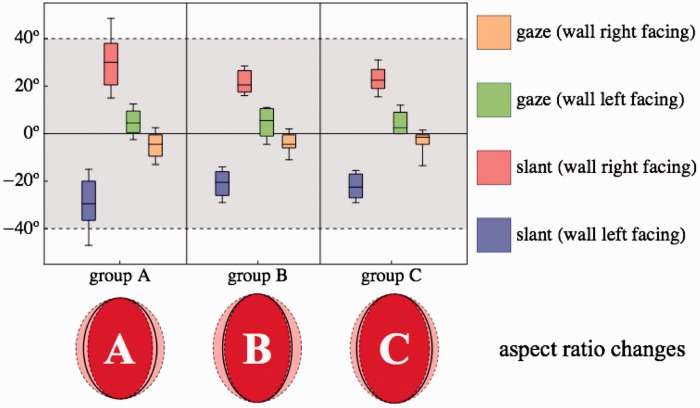
*Top*: Comparison of the main results for the three groups. Colors as in Figure 12 (left). The top and bottom of the gray region indicate the average slant simulated by the graphics. All groups underestimate these, albeit by different amounts. The whisker-box graphics indicate the quartiles and the 10% and 90% quantiles. The group of naïve observers has perhaps a somewhat larger spread, but the difference is slight. All groups reveal the same pattern. *Bottom*: The ellipses visualize the aspect ratio changes for the three groups (median and quartile range). They are also very similar.

In Figure 17, we have collected the summary results for the three groups. The conclusion is evident in that the same pattern is repeated over all three groups. It neatly quantifies the anecdotal account that Lord Kitchener keeps looking at you (gazing and facing), even when viewed from the side. The aspect ratio change is hardly ever mentioned in the literature (Koenderink et al., 2004). It is also similar for all groups.

### Overall Discussion Experiments I and II

In this section, we summarize the main results that immediately follow from the initial analysis.

All groups yield equivalent results for the main effect. What is novel in our analysis are many refinements and details that allow one to speculate with more confidence about the origin of the effect. Some simple facts immediately gleaned from Figures 11 and 12 are that the effect is indeed very significant, but that the slant is seriously underestimated.

When the wall faces right, the expectation is for the gaze to go over your right shoulder, by an angle equal to the slant simulated by the graphics. This is not the case, the gaze is much closer to frontal, which is the conventional “effect.” One might guess that the effect would perhaps not be 100% and that Lord Kitchener might look slightly over your right shoulder. However, the opposite is the case. Lord Kitchener looks slightly over your *left* shoulder. Likewise, with the wall facing left, the gaze is over your right shoulder. We offer no explanation, but this finding probably reflects the “illusion” described by Wollaston in 1824.

Other relevant structures in the data derive from inter-individual differences as well as idiosyncratic uncertainties and even apparent contradictions in the introspective reports. We can do this because the reports were severely straitjacketed (Experiment I) or framed in terms of quantitative indications of slant or gaze directions (Experiment II). The fact that we have a large number of participants enables us to collect various statistically significant inferences.

The inter-individual differences in the effect of monocular stereopsis (Fact II.11) are fully to be expected in the light of many previous findings. That a fairly large fraction of the generic population might have weak or perhaps even no monocular stereopsis is hardly a surprise either. From considerable experience over an extended period, we estimate that perhaps a third of the generic population may lack a fully articulated monocular stereopsis for photographs and naturalistic art works (Koenderink & van Doorn, 1995, 2003; Koenderink et al., 1992, 1994; Koenderink et al., 2001, 2009, 2011).

The effects found in this study are perhaps best understood by speculating that *the presentations in visual awareness are composed in mutually distinct spatial frameworks*. The framework that has the frame is pictorial space, which has a third dimension, depth, whereas the framework that has the face, or the smiley, is the visual field, which lacks the third dimension. That the face would be put in the visual field makes some sense because there are no strong cues that would suggest the opposite. This is different for the frame, which changes periodically between a rectangle and a trapezoid. The changes of the face are preferably presented as a simple 2D deformation, whereas those of the frame are more economically presented as due to perspective in 3D. What is conceptually striking here is that the presentations can “split” the material they present between distinct frameworks and apply distinct cues to each.

## Final Conclusions

We investigated the familiar phenomenon of the uncanny feeling that represented people in frontal pose invariably appear to “face you” from wherever you stand. We deployed two different methods.

The stimuli included the conventional one—a flat portrait rocking back and forth about a vertical axis—augmented with two novel variations. In one alternative, the portrait frame rotates whereas the actual portrait stays motionless and fronto-parallel and in the other, we replace the (flat!) portrait with a volumetric object. These variations yield exactly the same optical stimulation in frontal view, but become grossly different in very oblique views. We also let participants sample their momentary awareness through “gauge object” settings in static displays.

From our results, we conclude that the psychogenesis of visual awareness maintains a number—at least two, but most likely more—of distinct spatial frameworks simultaneously involving “cue–scission.” Cues may be effective in one of these spatial frameworks but ineffective or functionally different in other ones.
